# Long-Lived Epidermal Cancer-Initiating Cells

**DOI:** 10.3390/ijms18071369

**Published:** 2017-06-27

**Authors:** Marina Youssef, Andrew Cuddihy, Charbel Darido

**Affiliations:** 1 Department of Medicine, Monash University Central Clinical School, Prahran, VIC 3004, Australia; marina1youssef@hotmail.com; 2 Division of Cancer Research, Peter MacCallum Cancer Centre, Grattan Street, Parkville, VIC 3052, Australia; Andrew.Cuddihy@petermac.org; 3 Sir Peter MacCallum Department of Oncology, The University of Melbourne, Melbourne, VIC 3052, Australia

**Keywords:** squamous cell carcinoma (SCC), *Grainyhead-like 3* (*Grhl3*), interfollicular epidermis (IFE), supra-basal epidermis, *Involucrin* (*IVL*), cancer-initiating cell (CIC), cancer stem cell (CSC)

## Abstract

Non-melanomatous skin cancers (NMSCs), which include basal and squamous cell carcinoma (BCC and SCC respectively), represent a significant burden on the population, as well as an economic load to the health care system; yet treatments of these preventable cancers remain ineffective. Studies estimate that there has been a 2-fold increase in the incidence of NMSCs between the 1960s and 1980s. The increase in cases of NMSCs, as well as the lack of effective treatments, makes the need for novel therapeutic approaches all the more necessary. To rationally develop more targeted treatments for NMSCs, a better understanding of the cell of origin, in addition to the underlying pathophysiological mechanisms that govern the development of these cancers, is urgently required. Research over the past few years has provided data supporting both a “bottom up” and “top down” mechanism of tumourigenesis. The “bottom up” concept involves a cancer stem cell originating in the basal compartment of the skin, which ordinarily houses the progenitor cells that contribute towards wound healing and normal cell turnover of overlying epidermal skin layers. The “top down” concept involves a more differentiated cell undergoing genetic modifications leading to dedifferentiation, giving rise to cancer initiating cells (CICs). This review explores both concepts, to paint a picture of the skin SCC cell of origin, the underlying biology, and also how this knowledge might be exploited to develop novel therapies.

## 1. Introduction

The incidence of skin cancer is rising alarmingly, with rates of non-melanoma skin cancers (NMSCs) being up to five times that of all other cancers combined [1,2]. Since 1960, there has been a 3–8% increase in the yearly incidence of NMSCs, with the highest rates seen in Australia and lowest rates in parts of Africa, correlating with skin type. Of these NMSCs, basal cell carcinoma (BCC) and squamous cell carcinoma (SCC) are the most common subtypes, accounting for approximately 80% of all newly diagnosed malignancies worldwide. While cancer dissemination is uncommon in BCC, the rates of skin SCC metastasis are significant, commonly spreading to local lymph nodes. Lymph node involvement correlates with poor prognosis; the 5-year survival rate stands at 25–40% [3]. An estimated 100,000 people are treated for NMSCs each year in the US, of which 20–30% are SCCs. Additionally, an estimated 2–3 million new cases of NMSCs are identified annually worldwide. SCC rates are especially high in patients being treated for additional malignancies, such as with the use of B-RAF inhibitors to treat melanoma [4], or in patients that are immunosuppressed. Of particular susceptibility to SCC development are those also undergoing organ transplantation, due to the immunosuppressive regimen used to prevent graft-versus-host disease [5].

Despite the increase in incidence of NMSCs and the poor prognosis attached to some of them, the impact of NMSCs remains under-recognised. Together with BCCs, SCC treatments present the largest economic burden of all cancers, costing in excess of AU$300 million annually in Australia alone [5,6]. Treatments, which include electrodessication, curettage, radiotherapy, cryosurgery and topical medications, are often non-specific. Despite the available measures of combatting primary cancer growth, the high frequency of recurrence of SCC at corresponding or nearby locations to the primary lesions creates substantial challenges to the management of the disease. Furthermore, clinical investigations have suggested that recurrent SCC correlates with increased metastasis and poor prognosis [7]. The genomic landscape of recurrent versus primary tumours is not well understood, but given the well-established heterogeneous nature of SCC, recurrent tumours may share the same characteristics as the original tumour, or exhibit a completely different phenotype, and therefore, a different response to treatment. Genomic analyses in mouse models have shown that metastatic tumours share the same repertoire of mutations as the original primary tumour, but have a few additional genetic modifications that presumably add to the aggressiveness of the cancer [8]. Therefore, an understanding of the underlying pathogenesis of SCC, with the identification of its cell of origin, is critical for developing targeted strategies for effective SCC treatment.

## 2. The Skin Epidermis

The mammalian skin comprises the epidermis; a stratified squamous epithelium, and the dermis; an underlying matrix-rich connective tissue. Together they form a barrier to exclude toxins and pathogens from the body system and prevent water loss [9]. Scattered throughout the skin are pilosebaceous units that contain sebaceous glands and hair follicles (HFs), surrounded by the interfollicular epidermis (IFE) [10].

The epidermis is comprised of a basal layer at the interface with the dermis, and several supra-basal cell layers that have morphologically distinct regions, namely the spinous, granular, and cornified layers. It is within the epidermis that adult IFE cells sequentially undergo proliferation, migration, differentiation, and cell death. Each of these stages can generally be visualised by the unique and well-defined expression of various keratin proteins. Terminal differentiation of epidermal cells is identified by the Involucrin (IVL) expression that commences when cells migrate from the basal layers and begin to enlarge and differentiate, but prior to them undergoing the cell–cell cross-linkage that contributes to epidermal structural integrity [11,12]. Disruption of the balance between these four physiological processes, which can be assessed through aberrant expression of keratin markers, is a hallmark of cancer.

Skin integrity is maintained through continuous renewal and repair of the epidermis, mediated by signalling cascades within stem cell (SC) and progenitor cell populations, which result in terminal differentiation of these self-renewing populations. While SC populations are distributed in both follicular and interfollicular compartments, they have distinct functions in epidermal homeostasis. The IFE is the source of SC whose role is in routine epidermal cell renewal in the absence of injury. The hair follicle (HF) bulge contains a reservoir of multipotent slow-cycling, label-retaining cells (LRC) that are activated during the anagen phase of hair growth. LRC are also activated after wounding to facilitate repair and regeneration of both the HF and epidermis [13]. The HF bulge SCs give rise to transit amplifying (TA) cells, which are committed progenitors that remain undifferentiated, subsequently dividing to expand the progenitor cell population whilst sparing continued SC division. A balance of proliferation and differentiation between stem and progenitor cell populations is essential for the homeostatic maintenance of continuously regenerating skin, with normal epidermal cell turnover occurring approximately every 2–4 weeks [14,15].

The renewal of the entire epidermis under normal conditions has been attributed to a single type of self-renewing progenitor cell [16], and it is postulated that disruption of its renewal mechanisms can lead to various skin diseases, including cancer. Following a similar paradigm, development of both BCC and SCC are argued to originate from a single type of self-renewing cancer-initiating cell (CIC) that is effectively either a stem or progenitor cell, which has acquired a mutation that “primes” or predisposes the CIC population to accumulate additional “hits”. The right combination of “hits” will see the CIC develop into a self-renewing cancer SC (CSC), leading to BCC or SCC [17]. The reason why these CICs are thought to originate from stem or progenitor cell populations, rather than differentiated cells, is because they are innately self-renewing and thus require only minor genetic alterations for transformation into CSCs.

## 3. Squamous Cell Carcinoma of the Skin

Insights into SCC development arising from CICs or CSCs have mainly come from carcinogenesis studies in mouse models, especially those that use the well characterised 7,12-dimethylbenz(a)anthracene/12-*O*-tetradecanoylphorbol-13-acetate (DMBA/TPA) protocol for carcinogenesis. This protocol utilises a two-hit principle: first, DMBA is applied to the skin to induce the first “hit” in the form of mutations, resulting in a CIC. This is followed by TPA to enforce activation of Protein Kinase C (PKC), which is the second “hit” that promotes tumour growth [18]. The DMBA/TPA protocol is commonly used to accelerate carcinogenesis in animal models with distinct genetic backgrounds, and as DMBA-induced mutations are known to involve the Ras family of proto-oncogenes, deduction of the degree of susceptibility of specific genetically-characterised mice to carcinogenesis is feasible [19].

DMBA/TPA-induced carcinogenesis employed on the epidermis demonstrates that cancer initiation is an irreversible event. Murine models that are administered TPA one year after the last DMBA treatment develop cancer without significant delay, in a similar fashion to mice that are immediately exposed to TPA after DMBA treatment [20]. The very short latency of tumour formation using this protocol, regardless of when TPA is applied, indicates that the underlying DMBA-induced CICs are long-lived, slow cycling cells that fail to disappear over time [21]. This is striking in the context of epidermal cancers, as the nature of the epidermis is one of constant renewal and shedding of cells, together with the continued cycle of growth and regression of the HFs.

Elegant studies have largely focused on the HF bulge SCs, due to their longevity and their seemingly endless capacity towards cellular expansion. This was achieved by restricting Ras mutations to this specific cell-layer compartment, resulting in a high malignant capacity associated with mutant *Harvey-Ras* (*H-Ras*) expression in HFs. However, additional mutations in the *p53* tumour suppressor gene are required for these cells to give rise to SCCs. The addition of this genetic signature to slightly more differentiated TA cells arising from the bulge HFs does not lead to tumourigenesis [22]. This is supported by research using *Shh-*Cre^+^ mice to specifically express a mutant form of the *Kirsten-Ras* oncogene homolog with a glycine to asparagine mutation at codon 12 (*KRas^G12D^*) in TA cells, which fail to initiate papilloma formation, in contrast to HF-specific expression of the KRas^G12D^ mutant using *K15-*Cre^+^ mice, where papillomas frequently manifest [23]. In a similar vein, it has been shown that constitutively active Smoothened (*Smo*) in long-term resident progenitors found in the IFE and infundibulum correlates with BCC formation, whereas *Smo* mutants do not cause BCC in HF bulge SCs [24].

Kras^G12D^-induced mutations in HF SCs lead to dysregulation of both mitogen activated protein kinase (MAPK) and protein kinase B (AKT, also known as PKB)-mouse target of rapamycin (mTOR) pathways in this compartment. The resulting papillomas arise from keratinocyte hyperproliferation and cellular dedifferentiation. However, KRas^G12D^ mutations in TA cells have no impact on the MAPK or AKT-mTOR pathways [23]. The absence of an abnormal phenotype in TA cells suggests that mutant oncogene expression alone is not sufficient to generate a hyperproliferative and premalignant cell compartment with long-term renewal potential that could initiate cancer development. These results would indicate that the types of cells in which specific oncogenes are activated, whether multipotent SCs or differentiated or more terminally differentiated, determine the capacity for tumour development.

Expression of the H-Ras mutant in the IFE leads to benign papillomas, although they are rarely malignant, unlike in HF SCs where H-Ras gives them a highly malignant potential [25,26]. Likewise, the formation of malignant papillomas in the IFE is prominent following KRas^G12D^ mutant expression, indicating that squamous tumour formation is not restricted to the HF bulge [23]. However, constitutive Ras oncogene activation on its own does not appear to be sufficient to induce tumourigenesis within the IFE, and is dependent on continued administration of tumour promoters, or on additional genetic events. Concurrent loss of functional p53 in KRas^G12D^ expressing SCs establishes conversion to malignancy [23], and gain-of-function of p53^R172H^ mutations in this context confers a poorer prognosis [27]. Both carcinogen- and genetically-induced mouse skin SCC show recurrent mutations in Ras family, with copy number alterations of the *p53* gene [8]. In the absence of *Ras* mutations, loss of p53 can lead to hyperplasia and the appearance of markers of epithelial–mesenchymal transition. SCCs in this model only form following the induction of a second “hit”. Both the nature of the CICs and their specific oncogene activation, in addition to the contribution of the cell microenvironment, drive cancer progression, characterising the resulting tumour type and defining its malignant potential [28,29].

Despite the increasing evidence pointing to HF SCs as being the cells of origin of SCC, immunophenotyping studies aimed at characterising SCC samples have identified markers of IFE SCs (α6-Integrin^+^) in addition to HF SCs (CD100^+^, K15^+^ and K19^+^). This suggests that SCC-forming populations arise from both HF SCs and IFE SCs, although the relationship between the two populations in the pathogenesis of SCC is not well understood [30]. To dissect the contributions of these two populations of SCs to tumourigenesis, removing the IFE SC compartment while maintaining the HF SC compartment led to a reduction in the number of papillomas and SCCs compared to wild type mice, following wounding and subsequent administration of the tumour promoter, TPA [31]. The results of this, as well as other studies, suggest that SCC-initiating cells arise from slow-cycling populations in both the HF and the IFE, and the two SC populations function independently of each other [22,23,32].

## 4. Dedifferentiation in Epidermal Tumourigenesis

Although it has been shown that HF and IFE SCs are a source of CICs, acquisition of genetic modifications that lead to differentiated cell populations becoming more SC-like alone have been shown to be insufficient for malignant transformation. This suggests that the capacity for malignant transformation depends heavily on the specific cell population that acquires the specific mutation(s). Therefore, it is worth noting that the HF and IFE SCs are not the sole source of CICs. More differentiated cell populations can also undergo malignant transformation as a result of somatic mutations. For example, activation of Wnt signalling in the antral glands of the differentiated compartments results in adenoma formation in the mouse intestine, and these tumours were found to express high levels of the intestinal stem cell marker Lgr5 [33]. This data indicates that differentiated cells adopted a “progenitor-like” signature, which may highlight dedifferentiation as a potential tumour initiation process.

In the epidermis, benign papillomas, rather than malignant tumours, readily form in response to oncogenic mutations occurring in committed cells, similar to what occurs with TA cells. For example, when constitutively activated Ras mutants are expressed under control of the K10 promoter, which is activated in early differentiated supra-basal cells of the IFE, the result is benign papilloma formation with no malignant phenotype [34,35]. It is only upon concurrent overexpression of α_6_β_4_-integrin in the suprabasal epidermal layer that the frequency of papilloma formation increases following DMBA/TPA treatment [36]. This suggests that cells that have left the SC compartment and are undergoing differentiation are at least capable of developing benign tumours under the right conditions, usually requiring specific additional transforming events, a second or even third “hit” on top of the well-characterised Ras mutants, to facilitate malignancy.

More recent research further supports the notion that non-SC populations are in fact capable of initiating SCC formation. In vitro studies using viral vector transformations demonstrated that malignant transformation was evident in cells that have left the SC compartment, in spite of their more differentiated status [37]. Overall, though, compared to cells in the SC compartments, malignant conversion of skin papillomas arising from more differentiated cells to carcinomas is a relatively rare event that is characterised by alterations in the expression of markers including transforming growth factor-β (TGF-β) [38], Keratin-13 [39] and α_6_β_4_-Integrin [40]. Benign tumours that are at risk of malignant conversion are primarily derived from cells located within the HF, although the nature of the CIC remains the major determinant of malignant potential.

## 5. Grhl3 Is Expressed in the Differentiated Supra-Basal Compartment of the Epidermis

Research findings have since implicated the *Grainyhead-like 3* (*Grhl3*; also known as *Som*) gene in SCC formation arising from differentiated cells. This gene, one of three mammalian homologs of the *Drosophila grainyhead* (*grh*) gene, has been a focus of recent studies for its role in development and homeostatic maintenance of the skin barrier [41]. GRHL3 is a transcription factor mediating the expression of multiple target genes including structural proteins and lipid metabolising enzymes (e.g., Transglutaminase 1; TGase1) that regulate epidermal terminal differentiation and barrier formation [15,42].

Importantly, expression of GRHL3 is largely restricted to the somatic ectoderm/epidermis and is excluded from the progenitor-rich basal cell layer [42]. Heterozygote mice have no apparent phenotype when compared to controls, however, complete *Grhl3* knockout (KO) mice do not survive postnatally due to the downstream loss of TGase1 expression, which is responsible for the cross-linking of the protein and lipid matrix that form the epidermal barrier. This loss of TGase1 consequently causes aberrant epidermal barrier formation [41,43]. Epidermal acidification, homeostatic maintenance and preservation of the stratum corneum organisation are therefore perturbed, leading to pup dehydration and death immediately following birth [41,44]. GRHL3 is also involved in wound repair regulation through the transcription of another target gene, RhoGEF19 [45]. Loss of GRHL3 leads to downregulation of RhoGEF19 expression, resulting in the disruption of planar cell polarity, as well as defects in directional cell movement to heal embryonic wounds [46].

*Grhl3*-deficient embryos notably exhibit a cellular hyperproliferation response that is intrinsic to the epithelial cell, and not as a homeostatic response to aberrant barrier formation [43]. These epithelial cells demonstrate a loss of cell–cell contact inhibition, and an increase in proliferation as shown by elevated proliferating cell nuclear antigen (PCNA) marker expression. Furthermore, the mitotic cells that usually reside towards the basal epidermal layer, where the HF and IFE SCs as well as the TA cells are found, become increasingly prominent in the supra-basal layers, promoting a tumourigenic environment [47]. Pseudo-tumours form from these supra-basal cells when cultured in vitro, demonstrating their potential to become carcinogenic given the right microenvironment [47].

Interestingly, regression of the epidermal barrier does not occur in adults when *Grhl3* is deleted using Cre expression under the epidermal-specific *K14*-promoter (*Grhl3^∆/−^/K14Cre^+^*). This Cre recombinase is active from postnatal day 1 (P1) onwards, and thereby deletes Grhl3 expression efficiently after birth. Deletion of *Grhl3* at P1 does not lead to barrier defect nor to death from dehydration. This suggests that although Grhl3 is essential for barrier establishment, it is not required for crucial maintenance of the epithelium [47]. Recent data indicated that another Grhl gene, *Grhl1*, is compensating for the loss of *Grhl3* in adulthood, accounting for the ability of these conditional KO (cKO) mice to maintain an intact skin barrier [48].

## 6. The Role of Grhl3 in Skin Hyperplasia

*Grhl3*-deficient embryos exhibit keratinocyte hyperproliferation [47], in addition to aberrant wound healing [41] and fragile skin [49], suggesting that loss of GRHL3 interferes with the normal differentiation program, leading to an excess of cellular proliferation. Altered cellular differentiation and proliferation is a common characteristic of cancers whereby malignant cells, in addition to increased survival, also demonstrate dysregulated cell cycle progression [50,51]. On the other hand, aberrant epithelial growth, or dysplasia, is an early sign observed prior to tumour formation, such as following human papilloma virus (HPV) infection in, for example, the context of cervical cancer. These dysplastic epithelial cells, due to their accelerated growth and proliferative capacity, are more susceptible to acquiring other genetic changes, predisposing them to cancer development [52].

The *Grhl3*^∆/−^/*K14-Cre*^+^ cKO mice show mild hyperplasia and impaired terminal differentiation, although they lack defects in the homeostatic maintenance of the epidermal barrier seen in embryonic *Grhl3* KO mice [53]. Interestingly, however, these cKO mice develop more pronounced epidermal hyperplasia in regions of chemical injury, which correlates with increased expression of keratin 6 (K6), a marker for both the injury response and aberrant differentiation of keratinocytes [54]. Direct mechanical injury to the epidermis also increases K6 expression, together with keratin 10 (K10) [34,53], an early marker of terminal differentiation. In the normal response to injury, GRHL3 mRNA expression markedly increases together with increased GRHL3-expressing cells, indicating that Grhl3 is an important factor for the epithelial wound repair response [53]. Taken together, these results suggest that although GRHL3 is not necessary for maintaining the skin barrier after birth, it is highly involved in postnatal epidermal repair, particularly following chemically-mediated damage that leads to an inflammatory response, thereby providing a link between repair mechanisms, inflammation and keratinocyte proliferation.

## 7. Grhl3 Functions as a Tumour Suppressor against SCC

C57Bl/6 mice are well known for their high resistance to chemically induced tumourigenesis, and therefore, their capacity to form tumours following administration of the well-characterised DMBA/TPA carcinogenesis protocol is low [47]. *Grhl3^∆/−^/K14Cre*^+^ mice generated on a C57Bl/6 background readily develop multiple papillomas, keratoacanthomas (KAs) and SCCs. These mutant mice have an increase in both the frequency of papilloma formation and their progression to malignancies, with hyperplasia additionally seen in the epidermis surrounding these tumours following DMBA/TPA administration, reflecting the importance of GRHL3 in maintaining a tumour-free environment within the epidermis. Furthermore, when mice lacking Grhl3 are left to age without DMBA/TPA administration, they all develop epidermal hyperplasia and spontaneous papillomas, some of which eventually progress to SCC. This demonstrates that GRHL3 loss alone is sufficient for tumour initiation, and that its loss in the epidermis provides a potent stimulus for the development of spontaneous and chemically-induced aggressive skin SCC [47]. More recently, GRHL3 has been identified as a critical tumour-suppressor in head and neck SCC (HNSCC), a heterogeneous cancer associated with poor survival outcomes [55], and where loss of floxed *Grhl3* using *K14*-driven Cre leads to HNSCC development in the oral cavity of these mice [55].

**Figure 1:**
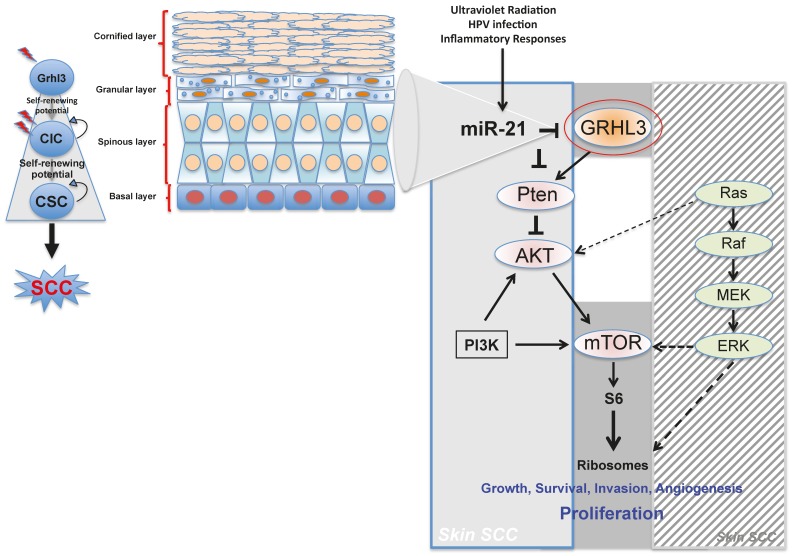
Schematic model proposing a differentiated cell as the cell of origin of skin squamous cell carcinoma (SCC). Cancer growth of Grhl3-deficient cells is dependent on the miR-21/GRHL3/PTEN/AKT/mTOR signalling pathway in skin SCC. **Middle** and **right** panels: GRHL3 (highlighted by a red circle is a key tumour suppressor preventing the onset of skin SCC arising from differentiated epithelial cells. Normally GRHL3 (highlighted in red circle) stands at the apex of a tumour suppressor pathway encompassing PTEN. When miR-21 is upregulated in response to ultraviolet radiation, HPV infection or inflammatory responses, it inhibits expression of *GRHL3* and *PTEN*, both directly and indirectly. Normally PTEN itself inhibits AKT activation driven by the PI3K pathway. Loss of PTEN leads to dysregulated activation of AKT and mTOR complex to activate S6 kinase, which stimulates ribosome biogenesis to facilitate the increased metabolic needs of cancer cells. The RAS oncogene is presumed to activate both AKT and MAPK pathways, leading to direct and indirect (via mTOR) increase in ribosome biogenesis; **Left** Panel: Loss of GRHL3 is thought to lead to a cancer-initiating cell (CIC) with self-renewing potential as indicated by the curved arrows. Further oncogenic “hits” induces a cancer stem cell (CSC) with self-renewing potential, which is ultimately the source cell for SCC.

Although experiments have clearly shown that mutations in members of the Ras family of proto-oncogenes play a role in development of SCC, and Ras mutations are prominent in about 30% of all human cancers, they actually occur at a relatively low frequency (10–20%) in human SCCs [54,56]. GRHL3 levels, on the other hand, are reduced in the majority of primary human SCCs with up to 90% reduction in expression when compared to adjacent, normal epidermis [47]. The high frequency of altered GRHL3 expression in human SCC compared to *Ras* mutations suggests that investigating the consequences of GRHL3 loss may better help understanding of the pathogenesis of SCC, as well as identifying rational therapies. Our laboratory has identified downregulation of the phosphatase and tensin homolog (*Pten*) gene arising from the loss of Grhl3 [47]. *Pten* a potent tumour suppressor gene, is a target gene of GRHL3 that catalyses the conversion of phosphatidylinositol (3,4,5)-trisphosphate (PIP3) to phosphatidylinositol (4,5)-bisphosphate (PIP2), leading to repression of the phosphoinositide-3-kinase (PI3K) signalling pathway. Loss of Pten as a result of reduced GRHL3 expression consequently leads to increased activity of 3-phosphoinositide-dependent protein kinase-1 (PDPK1) and AKT serine/threonine kinases, promoting cell survival, cell cycle progression, angiogenesis, cellular metabolism and growth of epidermal cells, which are all phenomena that are observed in multiple cancer types [57,58]. In addition, SCCs from *K14*-Cre^+^ cKO mice, which have constitutively active PI3K, do not harbour any mutations in H-, K-, and N-Ras [47], confirming that oncogenic pathways associated with RAS and PTEN are mutually exclusive in skin SCC [59] (Figure 1).

Mice that are heterozygous for the *Pten* locus (*Pten*^+/−^) have similar characteristics to those with Grhl3-deficient epidermis, developing hyperkeratosis (a thickened epidermal layer). Additionally, mice with a complete loss of *Pten* (*Pten^−/−^)* and a subset of *Pten*^+/−^ mice spontaneously develop SCC as they age [47,59]. Partial loss of *Pten* confers both an increase in the number of papillomas following DMBA/TPA application, as well as increased tumour progression [60]. Interestingly, carcinomas resulting from DMBA/TPA treatment of *Pten*^+/−^ mice seldom have the characteristic *H-Ras* mutation that is typically observed following DMBA treatment. Instead, the mice exhibit loss of heterozygosity (LOH) at the *Pten* locus, effectively becoming similar to *Pten*^−/−^ mice. These carcinomas effectively recapitulate what is observed in SCCs derived from *Grhl3* cKO mice, including reduced MAPK and increased PI3K/AKT signalling [47,59]. These findings, along with the fact that *Pten* is a direct transcriptional target of GRHL3, firmly places PTEN downstream of GRHL3 and that loss of either gene drives the malignant conversion of papillomas. Thus, the GRHL3–PTEN axis functions as a critical tumour suppressor pathway to prevent the onset of skin SCC.

Our laboratory also uncovered a novel proto-oncogenic pathway that drives formation of SCC in human skin. MicroRNA-21 (miR-21) is found to be overexpressed in many human SCC cell lines, and directly binds to the GRHL3 3′-untranslated region (3′-UTR), leading to downregulation of GRHL3 expression. This in turn leads to downregulation of *Pten* and increased PI3K activity, as described above [47]. Inhibition of miR-21 in human SCC cell lines rescued GRHL3 levels and normalised the downstream PI3K/mTOR signalling. Altered PTEN and RAS pathways are both evident in SCCs although, as mentioned previously, have been shown to be mutually exclusive pathways [59].

Interestingly, loss of Grhl3 in the oral epithelium of mice does not perturb the PTEN/PI3K/Akt/mTOR signalling, and rather leads to loss of expression of another GRHL3 target gene, glycogen synthase kinase-3β (*Gsk-3β*); which normally phosphorylates the c-MYC oncogene on threonine-58 (T58), marking it for ubiquitination and degradation, thus helping regulate c-MYC levels and activity. The reduction of *Gsk-3β* expression as a result of *Grhl3* loss results in the stabilisation and accumulation of c-MYC, and correlates with the formation of aggressive HNSCC [55].

## 8. The Dual Origin of Skin SCC

While tumours readily develop in mice following DMBA/TPA treatment, conditional deletion of floxed *Grhl3* in the differentiated cell-rich suprabasal epidermis using a tamoxifen-inducible Cre recombinase, driven by the *IVL* promoter (*IVL–Cre–ERT2*), confers an increased susceptibility to tumour formation similar to that seen with *K14Cre Grhl3* cKO mice. These inducible *IVL-Cre* driven *Grhl3* cKO mice develop more tumours and at earlier time points than controls exposed to the same carcinogenesis protocol. This is interesting since the nature of tumour cell development is to revert towards a dedifferentiated state, thus becoming more like upstream progenitor-type cells, which are more susceptible to malignant conversion, as has been observed in hepatocellular carcinoma for example. Regulation of progenitor cells occurs through the human exosome, specifically the EXOSC9 subunit. EXOSC9 maintains this cell compartment through promoting Grhl3 mRNA degradation within the stem cells, thereby preventing premature epidermal maturation through the expression of this transcription factor, and instead promotes self-renewal [61]. At least two distinct histological domains form from suprabasal SCC tumours; the outer portion of the tumour resembles well differentiated cells, whilst the core is poorly differentiated, a feature often correlated with tumour aggressiveness [62].

In keeping with this hypothesis, *Grhl3*-deleted mice using both *K14*-Cre and *IVL*–Cre are expected to develop SCC equally, and this is supported by preliminary data showing that SCCs formed in *K14*-Cre cKO mice have complete loss of *Grhl3* within the tumour, unlike the surrounding oral epidermis that still contains residual *Grhl3* expression [55]. Self-renewal markers including Sox-2, Oct-4, c-Myc and Nanog (unpublished data) should determine whether the mechanism of skin SCC formation corresponds to the previously characterised constitutive activation of the PI3K/mTOR/AKT signalling pathway or to an alternative activation of self-renewal pathways. Insights will warrant supportive evidence towards the possibility of a non-SC origin of SCCs.

## 9. A Differentiated Cell Could Be the Cancer Cell of Origin?

Epithelial-to-mesenchymal transition (EMT) has most commonly been described in the context of embryogenesis and development, whereby epithelial cells transition to a more primitive mesenchymal phenotype. These mesenchymal-like cells acquire properties of multipotent SCs, with a gain in SC marker expression, as well as invasive and migratory abilities, in addition to losing their cell–cell adhesion capabilities and cell polarity. Furthermore, EMT has been used to explain tumour development and invasion because mesenchymal cells possess many similar phenotypes as tumour cells. As this transition is able to create cells that have SC-like properties, it is possible that loss of Grhl3 may lead to induction of an EMT in differentiated suprabasal epithelial cells, thus facilitating the tumourigenic properties observed.

Ectopic expression of Twist and Snail in immortalised human mammary epithelial cells can lead to EMT [60]. Twist is a transcription factor whose targets are genes such as *PAR1*, which are known to confer invasive, migratory and SC-like properties, while Snail is a suppressor of E-cadherin, which is thought to be a suppressor of metastasis. The mesenchymal-like cells that arose from the mammary epithelial cells not only displayed fibroblast-like properties, but also exhibited properties that were associated with both normal and neoplastic mammary SCs. This adaptation therefore provides a potential pathway whereby differentiated cells can develop the SC-like capacity of CICs.

**Figure 2:**
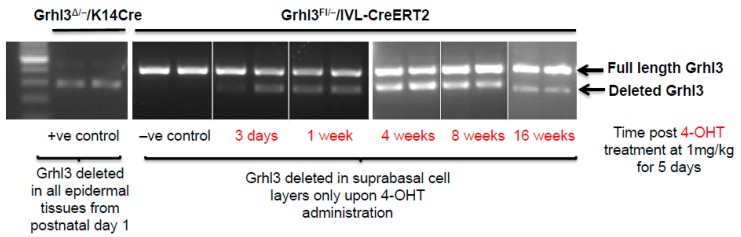
Genomic polymerase chain reaction (PCR) showing *Grhl3* deletion in the epidermis of *Grhl3*^Δ/−^/*K14*-Cre (constitutive Cre expression) and *Grhl3*^FL/−^/*IVL*–CreERT2 4-hydroxytamoxifen (4-OHT) induced mice. (**Left panel**) is a positive control. Middle and right panels show deletion at 3 days; 1, 4, 8 and 16 weeks post 4-OHT (as indicated in red) induction of involucrin (*IVL*)-Cre, demonstrating a similar ratio of deleted cells (Δ) at 1 and 16 weeks. This suggests that deletion of a *Grhl3* floxed allele (*Grhl3^FL/–^*) generates long-lived *Grhl3* deleted cells (*Grhl3^Δ/–^*) in suprabasal layers of the epidermis (normal tissue renewal is 2–4 weeks).

Despite the turnover rate of epidermal cells approximating 2–4 weeks [15], tamoxifen-inducible *IVL*-Cre (*IVL*-Cre-ERT2) mice, which delete floxed *Grhl3* in suprabasal cells, demonstrated that the Grhl3-deficient cells can be maintained within the mouse epidermis for up to 16 weeks following the beginning of the 4-hydroxytamoxifen (4-OHT) induction protocol in normal skin (Figure 2). Constitutive *K14-*Cre cKO mice, which show efficient Grhl3 deficient cells, were used as positive controls. In this case, the initial loss of Grhl3 leads to perturbation of the normal proliferative and differentiation capacity of the suprabasal cell population. Additionally, the long “dwell time” of these pre-malignant Grhl3 deficient cells means that there is more time for them to acquire the additional “hits” that leads to malignant conversion and SCC.

Although these findings have thus far indicated that Grhl3-deficient differentiated cells may be the cells of origin of SCC, an elegant study has identified a small population of IVL-expressing cells in the basal cell compartment by lineage tracing [63]. As these cells are progenitors, it is possible that the prolonged persistence of Grhl3-deficient cells observed through to 16 weeks in 4-OHT-treated *IVL*-Cre-ERT2+ mice could be due to the survival of these long-lived basal cell progenitor populations rather than the longevity of differentiated cells. Previous research has shown that approximately 10% of all basal cells exhibit expression of differentiation markers, which may indicate that the cells are committed to leaving the basal cell compartment for differentiation [64]. Further work comparing partial and complete loss of Grhl3 under the *IVL*-Cre promoter will address whether activation of these cell populations contributes to long-lived Grhl3 deficiency.

## 10. Conclusions and Perspectives

Increasing evidence in the literature supports the idea that differentiated cells have a cancerous cell-of-origin potential. Under specific genetic circumstances, these committed cells can revert to a CIC status with a longevity advantage, giving rise to cancer if excessively exposed to tumourigenic events—including HPV infection, inflammatory signalling and the exposure to sun ultraviolet radiation (Figure 1).

Lineage tracing and quantitative clonal analysis using Cre mouse models that are driven by promoters of differentiation genes such as *IVL* [63], but also by promoters which are more tightly restricted to the differentiated compartment, would confirm the identity of the Grhl3-deficient CIC in the presence of DMBA/TPA. Following confirmation of the identity of the cell of origin, the ultimate aim will be to characterise it in the context of human SCC, in order to develop targeted therapies for effective treatment. Chemotherapeutic agents including cisplatin, ifosfamide, and docetaxel, have shown some efficacy in skin and HNSCC, and although combination therapy is also effective, toxicity resulting from this treatment is substantial [65]. The current scope for SCC treatments, which include curettage, electrodessication, excision or cryosurgery, is non-specific and thus carries a high economic burden.

A substantial amount of literature recognises that even among CSCs, there lies a great deal of heterogeneity. Studies on HNSCC, for example, have identified heterogeneity within tumours through differential expression of the CD44 cell surface glycoprotein, where cells expressing this factor also had increased expression of the BMI-1 gene that has been shown to play roles in self-renewal and tumourigenesis [66]. Moreover, tumour microenvironment can also influence heterogeneity via non-genomic factors, including expression of metabolites and gradients of various cytokines and growth factors that also have an impact on the effectiveness of drugs used to treat the tumours. For example, a recent study has shown that differential TGF-β signalling within SCCs influences tumour drug responses, including resistance to cisplatin, which is one of the most widely used anti-cancer drugs [67]. Further investigations manipulating factors derived from non-epithelial tissues of the microenvironment would shed light on how the microenvironment impacts SCC development, and whether targeting those factors would have a beneficial effect against CSCs to reverse their resistance to therapies.

By establishing a genetic signature for various SCCs (for example, loss of *Pten* versus loss of *Gsk-3β*), and understanding how different signatures respond to different treatments, patients can be stratified according to their tumour genotype in order to receive drugs that are specific to their tumour status. Additionally, therapies aimed at the CIC, and subsequently, the CSC, will enable elimination of potential resistant tumour cell populations. In the case of the CIC, the benefits of eliminating a population of pre-malignant cells before they develop into full-blown tumours would be enormous. This is because the slow-growing CIC and CSC populations are intrinsically more resistant to conventional therapy, and can eventually lead to a refractory scenario where the tumour no longer responds to treatment. Thus, eliminating the CIC and CSC populations will potentially allow the bypassing of acquired drug resistance in response to therapy for SCCs of the skin, and also for more heterogeneous and aggressive SCCs, such as HNSCC.
